# Piloting a Spanish-Language Web-Based Tool for Hereditary Cancer Genetic Testing

**DOI:** 10.3390/curroncol30090606

**Published:** 2023-09-12

**Authors:** Deborah Cragun, Gretter Manso, Stefania Alastre Arcusa, Brenda Zuniga, Julie Dutil, Marcia Cruz, Tuya Pal

**Affiliations:** 1 College of Public Health, University of South Florida, Tampa, FL 33620, USA; 2 Department of Human Genetics, University of Miami Miller School of Medicine, Miami, FL 33124, USA; g.manso@med.miami.edu; 3 Moffitt Cancer Center, Tampa, FL 33612, USA; stefania.alastrearcusa@moffitt.org; 4 Vanderbilt–Ingram Cancer Institute, Nashville, TN 37232, USA; brenda.i.zuniga@vumc.org; 5 University of Puerto Rico Comprehensive Cancer Center, San Juan, PR 00936, USA; jdutil@psm.edu (J.D.); marcia.cruz1@upr.edu (M.C.); 6 Ponce Research Institute, Ponce Health Sciences University, Ponce, PR 00716, USA; 7 Department of Medicine, Medical Sciences Campus, University of Puerto Rico, San Juan, PR 00935, USA

**Keywords:** hereditary cancer, genetic counseling, cancer education

## Abstract

The delivery of hereditary cancer pre-test education among Spanish-language patients is impeded by the dearth of Spanish-speaking genetic counselors. To address this gap, we evaluated a web-based genetic education tool delivered in Spanish to provide information typically discussed during an initial genetic counseling session. Spanish-speaking patients with a personal or family history of cancer were recruited at two centers in Puerto Rico and through social media. A total of 41 participants completed a survey before and after viewing the tool to measure knowledge, attitudes, and decisional empowerment. A subset of 10 participants completed a virtual semi-structured interview to assess the usability and appropriateness of the tool. Paired t-tests were calculated to evaluate changes in knowledge and attitudes. A McNemar test assessed for decisional empowerment. Interview transcripts were translated from Spanish to English and inductively coded and analyzed. Results revealed significant increases in knowledge (*p* < 0.001), while attitudes about genetic testing did not change (*p* = 0.77). The proportion of individuals who felt fully informed and empowered to decide about whether to undergo genetic testing increased from 15% to 51% (*p* < 0.001). Qualitative data indicated that participants found the tool easy to use with informative and valuable content. Our findings suggest this Spanish-language tool is a user-friendly and scalable solution to help inform and empower many individuals to decide about cancer genetic testing, recognizing that others may still benefit from genetic counseling prior to testing.

## 1. Introduction

Approximately 5–10% of cancers are inherited due to a germline gene mutation. Identifying individuals with an inherited cancer-predisposing gene mutation enables strategies for early detection or cancer prevention, and results may influence cancer treatment in those with a cancer diagnosis [1]. Prior studies report low awareness and uptake of genetic testing services for inherited cancers among the Hispanic/Latino (H/L) populations in the United States [2], who comprise more than 18% of the population and represent the largest minority group. Despite the low awareness, studies suggest once aware, H/L individuals are interested in learning more about inherited cancer and testing [3]. However, access to these services by Spanish speakers is limited due to a multitude of barriers including a dearth of Spanish-speaking genetic counselors [4]. Consequently, most Spanish speakers within the continental U.S. receive genetic counseling through inadequate interpreter services and report problems with verbal and written communication during visits [5]. Although Spanish is the dominant language in Puerto Rico, there are no Master’s trained genetic counselors practicing in the territory, and the need for additional information has been reported as the most common barrier to genetic testing among cancer survivors in the territory [6].

Online educational tools have been proposed as a strategy to deliver pretest inherited cancer education [7], yet there remain limited efforts to evaluate Spanish language online educational tools. We previously developed an English version of an interactive web-based tool which significantly increased knowledge across different health literacy levels and increased the number of individuals who felt informed and empowered to decide about genetic testing [8]. Leveraging these efforts, we developed a Spanish version of the tool and measured the impact of the tool on (1) knowledge, (2) decisional conflict/empowerment, and (3) attitudinal values about testing. We also evaluated the tool’s usability and appropriateness among Spanish-speaking individuals.

## 2. Materials and Methods

This study was approved by the University of South Florida Institutional Review Board (STUDY001215). All participants were consented via a Spanish language consent form.

### 2.1. Development of the Spanish-Language Tool

A previously developed and tested web-based interactive tool designed in English to provide education about inherited cancer [8] formed the basis of the Spanish language tool. The tool was animated and designed to cover educational aspects of a hereditary cancer genetic counseling pre-test session. The core audio and visual content could be viewed in about 12 min with optional “learn more” content.

The tool is focused on hereditary cancers in general and includes 12 core (mandatory) slides with animation and audio focused on inheritance, cancer risks, types of test results, how results may or may not change medical management, testing benefits/limitations, insurance considerations, and the genetic testing process. There are an additional 22 optional slides with detailed content that relates to or expands on core content. Examples of additional content include an explanation of the difference between tumor versus germline testing, the concept of ‘best testable family member’, and gene-specific content on 17 inherited cancer-predisposing genes. Individuals can navigate the tool at their own pace using arrows to proceed to the next slides, go back to prior content, or click on the ‘learn more’ content. Based on the Participatory and Iterative Process Framework for Language Adaptation (PIPFLA) [9], the tool was translated to Spanish and back-translated to English to ensure accuracy., The translation was then reviewed and feedback was incorporated to reach consensus on the final translated version.

### 2.2. Participant Recruitment and Data Collection

The developed tool was tested among Spanish-speaking individuals living in the United States or territories who were 18 years or older, whose preferred language(s) for receiving health information was Spanish or both English and Spanish, and who had a personal and/or family history of any type of cancer. Participants were recruited through multiple strategies, including a flyer distributed through social media (Facebook, Instagram, and WhatsApp) and in person prior to their oncology or surgery clinic visits among patients seen at a cancer center in Puerto Rico. The flyers contained an email address and/or link that allowed participants to learn more information about the study. Individuals who met the study criteria were instructed to review the Spanish informed consent form, following which they were directed to complete a baseline survey to measure knowledge [8], decisional conflict/empowerment [10], and attitudinal values [8], prior to viewing the tool. Upon completion, participants were directed online to the educational tool. After viewing the tool, they were instructed to complete a final survey, which measured items in the baseline survey to assess for changes in measures. Participants who failed to complete all aspects of both surveys were excluded from the study.

Participants were asked on the post-tool survey if they would be willing to be interviewed to elicit additional feedback, and those who indicated willingness were invited by phone and/or email to participate in a recorded 60–90-min, individual interview to provide additional feedback. Prior to completing the interview, participants provided verbal consent to record. No personal health information was requested during the interview. Interviews were conducted in Spanish remotely using online screensharing software accessible from a desktop or tablet. Participants who completed an interview were given a $20 gift card.

### 2.3. Pre- and Post-Tool Survey Measures

The Spanish surveys used for this study were similar to surveys used to test the English version of the tool [8]. The pre-tool survey contained basic demographic and clinical questions as well as a previously validated single-item question [11], “How often do you have problems learning about a medical condition because of difficulty understanding written information?” Response options for this question ranged from 1 (never) to 5 (always) and served as a proxy measure for health literacy.

Both the pre- and post-tool surveys contained knowledge questions about hereditary cancer testing [8], the SURE checklist [10] to measure decisional conflict/empowerment, and 3 items to assess attitudinal values toward genetic testing [8]. Knowledge items consisted of accurate and inaccurate statements about topics addressed in the tool (e.g., inheritance, possible test results, extent to which laws protect against genetic discrimination, etc.). Response options for each item included ‘agree’, ‘disagree’, and ‘don’t know’. To calculate knowledge scores, 1 point was assigned for each correct answer they agreed with or each incorrect answer they disagreed with and scores were summed across the 9 items.

The four-item SURE checklist [10] was used with response options of ‘yes’, ‘no’, and ‘unsure’. These items assess whether individuals feel they have enough support and advice to make a decision, know the benefits and risks of the options, are clear which benefits and risks matter most to them, and are sure about the best choice for them. Participants who answered yes to all four SURE checklist items were classified as feeling fully informed and empowered to decide about genetic testing, while all others were classified as having at least some degree of decisional conflict about genetic testing.

Attitudinal values about genetic testing were collected through three 5-point, Likert-scale items to assess the extent to which individuals agreed or disagreed that they wanted genetic testing, thought it would be useful, and felt it was important for them. These three items demonstrated high inter-item reliability among Spanish-speaking participants who completed the pre-survey (Cronbach alpha = 0.89) and were averaged to create an ‘attitudinal values’ score ranging from 1 to 5 (higher numbers indicating more positive attitudes).

### 2.4. Qualitative Interviews

During the interviews, participants were asked questions using a semi-structured interview guide, and most participants observed the web-based tool while discussing its content. The interview guide was created in English and translated into Spanish. To ensure accuracy, the guide was then back-translated into English by a separate native Spanish speaker who was part of the investigative team. Questions elicited feedback about specific aspects of the tool’s visual aesthetics, audio quality, visual/design appeal, and recommendations for improvement. Given that one of the primary goals of the tool was to prepare individuals to receive results of genetic testing, we also asked participants how they would feel about the different types of test results for the purpose of assessing participants’ understanding of the results and whether they were able to accurately apply that understanding to their own situation. Interviews were adaptative in nature, and additional probes about the tool were added based on the feedback that was received from the first few participants. Sonix AI transcription software was utilized to transcribe the interviews in Spanish, and they were then translated into English using Google Translate and then verified by a native Spanish speaker by comparing them to audio recordings.

### 2.5. Quantitative Data Analysis

Data from the pre- and post-tool surveys were analyzed using SPSS Statistics (IBM corp, version 26). Mean scores and paired samples *t*-tests were calculated to evaluate pre–post changes in knowledge and attitudes. For any significant changes, Cohen’s d was also calculated to measure the effect size, where 0.2 is considered small, 0.5 is considered medium, and greater than or equal to 0.8 is considered large [12]. A McNemar test was conducted to evaluate changes in whether participants felt informed/empowered to decide about genetic testing before and after viewing the tool.

### 2.6. Qualitative Data Analysis

Qualitative data analysis of the interview transcripts was completed using Nvivo software. Translated interview transcripts were coded by a single member of the team using a priori and emergent codes related to constructive feedback about the tool’s aesthetics, additional information needed, preferences, recommendations for improvement, as well as understanding of the tool’s content (e.g., hereditary cancer and possible test results). Codes were revised with input from a second team member who reviewed the codes and example quotes for each. Codes relating directly to the tool were organized into a prior and emergent categories/subcategories, while codes related to participant’s understanding of possible testing outcomes were organized into themes using Braun and Clarke’s method of thematic analysis [13].

## 3. Results

### 3.1. Quantitative Survey Results

The pre-tool survey was accessed 109 times, but only 65 unique individuals completed any of the pre-survey questions. Of those 65, 41 (63%) also reviewed the tool and completed the post-tool survey. There were no statistically significant differences between those who did and did not complete the post-tool survey in terms of demographics, clinical information, or baseline scores. Table 1 includes demographic and clinical data for the 41 completers. Most participants were female and Caribbean Islanders with a family history of cancer. Mean pre- and post-tool knowledge scores significantly increased from 3.7 to 5 with a Cohen’s d of 0.56 indicating a medium effect size was obtained [12]. No changes were seen in genetic testing attitudes with a mean score of 3.10 before and 3.13 after (*p* = 0.77). Only 15% of participants felt fully informed and empowered to decide about genetic testing before viewing the tool, which increased significantly to 51% after viewing the tool (*p* < 0.001).

### 3.2. Qualitative Interview Results

A total of 10 participants completed an interview. For items specifically related to educational tool content, feedback from the semi-structured interviews was categorized into four main categories: aesthetics, content, comprehension, and general recommendations, with each category having several subcategories as shown in Table 2 (see Supplementary Material Table S1 for Spanish version). Specifically, for the aesthetics category, most participants found the tool to be visually pleasing and “dynamic”, the audio proceeded at an appropriate pace and the narrator’s voice was clear and understandable, although they commented that audio volume should be uniform throughout the tool. Regarding content, most participants indicated the tool was valuable to them based on the knowledge they gained both for themselves and for family members.

General recommendations included the development of ancillary materials such as flyers or pamphlets, additional information about insurance protections/discrimination and factors influencing the risk of sporadic cancer, enabling access to other populations such as the hearing impaired, and simplifying information wherever possible by reducing the reading level. Despite these suggestions, most participants expressed that the tool is adequate for general public use.

Several qualitative themes shown in Table 3 (see Supplementary Material Table S2 for Spanish version) emerged regarding participant’s understanding and emotions related to the content of the educational tool, including: testing benefits to family members, importance of cancer family history, and responses to types of test results (positive, negative and VUS). Many individuals clearly seemed to grasp the implications of possible test results. Generally, participants expressed they understood that genetic testing could lead to actionable findings with prevention and management guidelines and could also be beneficial for family members. Participants also voiced understanding the importance of family history to guide cancer screenings, even if they did not have a positive test result. Despite most responses demonstrating understanding of the information, Table 3 also includes misunderstandings that were identified.

Responses to how participants would feel if given a positive test result were grouped into three thematic areas: (1) an emotional response (e.g., feelings of concern and worry); (2) confusion about the meaning of a positive test result (e.g., equating it to being given a cancer diagnosis); and (3) actions to be taken based on learning about a positive test result (e.g., cancer risk management and family communication). Responses to negative results were grouped into two thematic areas: (1) lack of clarity about the meaning of the result in the context of a breast cancer diagnosis; and (2) understanding that negative results do not eliminate all cancer risks and ongoing screening is important. Finally, responses to VUS results were grouped into three thematic areas: (1) trying to differentiate it from a positive result, with one person failing to recognize that a VUS can be inherited and therefore present in the germline; (2) understanding that family history was still important to guide cancer risks and screening; and (3) reactions to VUS and negative results would be similar.

## 4. Discussion

To our knowledge, this is the first study to evaluate the efficacy of a Spanish genetic education tool focused on cancer genetics pre-test education [14]. Our findings demonstrate that Spanish-speaking participants’ knowledge and feelings of being informed and empowered to decide about genetic testing significantly increased after viewing the tool; however, all individuals did not achieve the same level of understanding of genetic testing information, suggesting that some individuals may benefit from additional counseling to address knowledge and decisional gaps. In addition, findings from a subset of participants who were subsequently interviewed suggest the tool is appropriate and acceptable among Spanish speaking individuals who have a personal or family history of cancer.

While a few prior Spanish language educational tools focused on hereditary cancer have previously been evaluated in the published literature [15,16], none to date have tested the efficacy of a Spanish-language tool that is intended to cover elements of pre-test education as we have. In fact, improvements in knowledge about hereditary cancer genetic testing demonstrated by participants after viewing the tool suggest its potential to supplement or replace pre-test genetic counseling. Furthermore, there is potential for the tool to reduce the length of the appointment or allow more time to discuss concerns and questions patients may have, such as emotional, social, and/or personal concerns and may provide an opportunity to clarify any misunderstandings [17].

While H/L populations have less awareness about genetic testing compared to Whites [18], there are still insufficient numbers of Spanish-speaking genetic counselors to meet population needs for Spanish-preferring patients [4,19]. Data suggest that once Spanish-preferring patients become aware of genetic testing, interest is high in these services both among young and older H/L populations [20,21]. Our tool provides a scalable solution that is not dependent on numbers of trained Spanish-speaking counselors. Prior studies have shown that the receipt of services through a translator among Spanish-preferring patients results in suboptimal care [22], including misinterpretation of information and less opportunity for shared decision making [23,24]. Specific areas that are particularly challenging are an explanation of VUS results, which are more common in H/L individuals undergoing genetic testing [25]. Data from our current study demonstrate enhanced understanding of content related to different types of test results. Furthermore, given inadequate interpreter services and problems with verbal and written communication that Spanish-preferring patients often experience [5,26], our tool has the potential to improve the quality of the genetic counseling visit. Moreover, the tool may ultimately prove useful for triaging those who may not need pre-test counseling. Specifically, if an individual feels fully informed and empowered to make a decision about testing and they have sufficient understanding after viewing the tool, they may not need further counseling, which would reduce the number of Spanish-preferring patients who would need to meet with a genetic counselor.

One of the purposes of the pre-test genetic counseling session is to be able to make an informed decision about testing and reduce decisional conflict. We saw a significant reduction in decisional conflict, with just over half of all participants feeling fully informed and empowered after viewing the tool. Similar to our findings when evaluating the English version [8], the tool did not alter attitudes nor persuade people to pursue genetic testing, as this was not what the tool was intended to do.

While there is potential for this tool to be useful among Spanish-preferring individuals, some feedback from the qualitative data is of use to further improve the tool, including enhancing audio quality, improving clarity of certain visuals, providing opportunities to access additional information, simplifying and clarifying terminology as much as possible, and expanding access through closed captioning.

The current study has several strengths, including the collection of both quantitative and qualitative data. Although this tool was tested in the continental U.S. and Puerto Rico, the potential impact of this tool is much broader given that Spanish is one of the most commonly spoken languages in the world. While this tool was not originally developed by native Spanish speakers and did not incorporate any cultural nuances, the information contained in this tool is based on American Society of Clinical Oncology guidelines for content to discuss prior to genetic testing for hereditary cancer. Furthermore, the tool was translated by a native Spanish speaker and was acceptable to the Spanish speakers in this study. Nevertheless, future research could further assess cultural acceptability among a broader range of Spanish speakers.

Despite several strengths, our study had a limited sample size, and most participants were Caribbean Islanders with high educational levels, which limits generalizability. Furthermore, our study was not designed to measure the impact of the educational tool on the subsequent genetic counseling process. Given our efforts were focused on evaluating our tool, comprehensive personal and family history information to determine indications for testing was not obtained. Finally, retention of knowledge over time and impact on testing uptake was beyond the scope of the current effort.

Findings from our study indicate that this Spanish-language web-based tool for hereditary cancer significantly increased knowledge and reduced decisional conflict pertaining to inherited cancer testing and care in a group of Spanish-speaking patients. Furthermore, the tool was well received overall and deemed appropriate and usable for Spanish-speakers. Our approach represents a promising scalable strategy to deliver informational content that is recommended to be discussed during a pre-test genetic counseling session focused on inherited cancer.

## Figures and Tables

**Table 1:** Demographic and clinical information for the 41 who completed the pre- and post-tool survey.

	*n* (%)
**Gender**	
Female	31 (75.6)
Male	10 (24.4)
**Age**	
18–29	3 (7.3)
30–39	3 (7.3)
40–49	11 (26.8)
50–59	12 (29.3)
60–69	9 (22.0)
70–79	3 (7.3)
**Preferred language of medical information**	
Spanish	31 (75.6)
Both equally	10 (24.4)
**Clinical cancer history**	
Never diagnosed	22 (53.7)
Previously diagnosed	4 (9.8)
Currently diagnosed	15 (36.6)
Has a family history of cancer	38 (92.9)
Genetic testing completed	11 (26.8)
**Family completed genetic testing**	
Yes	11 (26.8)
No	23 (56.1)
Don’t know	7 (17.1)
**Race/Ethnicity**	
Caribbean islander	35 (85.4)
South American	1 (2.4)
North America (U.S./Canada)	2 (4.9)
Central American	1 (2.4)
Other	1 (2.4)
Prefer not to answer	1 (2.4)
**Problems learning about medical conditions**	
Never	14 (34.1)
Rarely	13 (31.7)
Sometimes	13 (31.7)
Most of the time	1 (2.4)
**Educational level**	
High school not completed	2 (4.9)
High school completed	13 (31.7)
Some college	1 (2.4)
College graduate	11 (26.8)
Postgraduate degree	14 (34.1)

**Table 2:** Categorized feedback about the tool from semi-structured interviews with representative quotes.

	Subcategory	Quotes
Aesthetics	Visual Quality	“I loved the design of the presentation in terms of the visuals from beginning to end. I found it friendly, as if flashy. From the first time I saw it, that was my immediate impression, that it was eye-catching”
	Audio Quality	“Well, the quality could be a little better. The volume sometimes varied depending on some slides where the volume was higher and others were lower and quality was half and half”
	Narrator’s Voice	“It doesn’t bother me, it seems to me that she spoke clearly”
Content	Complexity or Unfamiliarity with Language	“If it’s the first time, it might be a little confusing or something, because the language wouldn’t be a normal thing for it, you know”
		“Obviously I also think that the person has to have a little, a little bit of knowledge of what genetics is because they’re going to see the term that maybe they’re not going to be very familiar with”
	Easy to Understand/Simple	“The presentation is extremely well written in terms of carrying in understandable language a lot of information that which is not so simple”
		“It seems to me that the information is quite complete and I like that the language is simple, even though the information it is giving is quite detailed information as to what the causes are and how they work”.
		“Yes, of course. Its information is clear and simple to understand”
	Value of Information	“I think one of the things I remember that I stayed with was that even though I’m a man, I have and have children, including a girl—I think was helpful for me to take the test because it can make an impact. You can give me information for my daughter, even though I am a man”.
		“This, which was good for me to be interested in that variant that obviously not right now has been studied, so it is not but, having seen the presentation helped me understand what the [VUS] meant…. I haven’t had the doctor’s appointment yet and have it next week but at least I could get an idea of what it was I was looking at when I saw the results. This I think… I think the presentation is extremely valuable”.
Comprehension	Understanding of Hereditary Cancer	“Well, as I understood hereditary [cancer] is less possible. Only 10%, right? It is less possible to get it by inheritance”.
		“I could put it in simple words, there is genetic information that we can inherit and could or could not develop, but that we are at risk is that we have some predisposition to give that genetic information, yes. As I said, it is a Russian roulette, we can have the gene and not get [cancer]. We can have it, and then get [cancer]”.
	Risk Perspective	“Well, because of my family history, I have to be a little more careful than other people have, even though my results of the specific genes that were studied, that are being studied came out negative. This, my family history in that case is the one that predominates so my risk is a little higher than normal”
General Recommendations	Mode of Delivery	“I think always that flyers and pamphlets, that kind of thing that is something that really helps with medical issues”
		“Look, if there was like an interview, one person [talking] with another, or probably a testimony, like, look, I came out negative and my mom came out positive, it was positive and look, and we took the test, and it turned out that I came out. Huh, uh, uh, do you understand me? So that same one that you explained, but that a person says it, understand, I assure you that I will stay [paying attention] longer”
	Additional Information Needed	“If anything, dig a little deeper into the issue of insurance, because I think that’s an area…I don’t know. Maybe if I were the only one who had it but I think that digging a little more to give more security to the people who do contemplate having these exams in the future to give you more assurance that they are protected”. “If second part says mutations may be caused by errors during cell division or may be caused by exposure to agents in the environment that damage DNA… although I don’t know if you’re going to explain what those agents are later, but maybe they’ll explain it later”.
	Access Barriers	“For example, …older people who are going to need a little [more] follow-up (in terms of understanding the concepts)”. “If someone like us sees it, [they will not] find barriers but for someone who cannot hear it, because it will be difficult to at least hear what the [narrator says] while reading I think not but that is taking into consideration that this community will access this type or this presentation”
	Endorsement For General Public Use	“I found it very educational, and it seemed like something that should be shared with more people, not only the people who participate in this study, because there are many times that sometimes even in television programs and that makes jokes about it, but that many times people do not understand what it means even the most basic concepts, of negative or positive” “It’s a good idea that another person before going through this process saw this presentation. It is very informative”.

**Table 3:** Themes from semi-structured interviews related to possible testing outcomes.

Themes	Exemplar Quotes	Interpretive Conclusions
Testing benefits family members	*“Those who come out with the positive result in the BRCA that we were talking about indicates that when the man, even if [they] don’t have it, that you take it from your mother or from someone, it can affect the little girl of mine that I have, who may have greater possibilities to get [cancer]”; “…the results help the family”.*	Suggests understanding that even if they never developed cancer, their children could be at risk if they were to inherit a mutation in a cancer-predisposing gene.
Importance of cancer family history	*This type of sporadic cancer, hereditary,* etc. *the genetic testing of mutations that when it comes out positive, when the VUS variant comes out, when it is negative, but always taking into consideration the family history of cancer until the end.*	Suggests understanding of the importance of family history in considering cancer screening, even without a positive genetic test result.
Responses to positive genetic test results	*“Destroyed. I would feel bad. I would feel like worried or very worried”*	Demonstrates an emotional response of concern or worry about the results which is appropriate.
	*“fight to have life and see how a proper recovery is possible”;*	Suggests a potential lack of clarity in the meaning of a positive test result and viewing a positive genetic test result similar to being given a cancer diagnosis.
	*“Well, I think pretty prepared, right? This in the sense of knowing what the next step is and in addition to discussing it with the primary care physician, sharing it with family members or progeny. My children and the moment I have them let them know this is already genetically proven or you have to be aware”.*	Demonstrates understanding of next steps based on positive results, including seeking prevention, speaking with doctors, and sharing the information with family members.
Responses to negative genetic test results	*“Confused with you because if I receive a negative and I am positive for breast cancer. Well, I do not know, on the one hand it can be. If I take this out, the negative genetic test and I have cancer, or if it comes out positive for cancer like would my daughters have this. I mean, like how?”*	Suggests a lack of understanding regarding the meaning of a genetic test result.
	*“Fantastic, great, awesome. I would be happy. I would feel more confident but that would not take away my desire to check myself every now and then”.*	Demonstrates understanding that negative results do not completely eliminate all cancer risks, and that prevention methods and check-ups would still be sought out.
Responses to VUS genetic test results	*“If it’s like what I’ve understood from the video, it’s that a random mutation occurred, so it’s not hereditary. It’s not something we inherited from mom or dad, but it was just a mutation that happened randomly in the body and then that genetic change was found in the test, but you can’t see, you don’t determine that it comes from dad or that it comes from mom”.*	Suggests trying to differentiate result from a positive result, yet misunderstanding that a VUS may or may not be inherited.
	*“Well either way there is uncertainty because this like you have to get carried away by the family history of cancer as well. No, it’s not that you’re already excluded or that you’re 100% exonerated”.*	Demonstrates understanding that increased risks of cancer may exist based on family history; thus, seeking clinical advice and screenings are still important
	*“I know I would feel the same way as if I had tested negative”*	Demonstrates reaction to VUS and negative results would be similar which is appropriate given that most VUS results are ultimately reclassified as negative.

## Data Availability

Data are available upon request.

## Supplementary Materials

The following supporting information can be downloaded at: https://www.mdpi.com/article/10.3390/curroncol30090606/s1, Table S1: Categorized feedback about the tool from semi-structured interviews with representative quotes in Spanish; Table S2: Themes from semi-structured interviews related to possible testing outcomes with quotes in Spanish.
