# Supplementary material for: Piloting a Spanish-Language Web-Based Tool for Hereditary Cancer Genetic Testing
Source: Curr Oncol. 2023 Sep 12;30(9):8352–62. doi: 10.3390/curroncol30090606 (PMC10529239; doi:10.3390/curroncol30090606)
Supplement: Supplementary file 1 [file curroncol-30-00606-s001.zip › curroncol-2533512-supplementary.pdf]

Supplemental Material

Table S1. Categorized feedback about the tool from semi-structured interviews with representative quotes in Spanish.

	Subcategory	Quotes
Aesthetics	Visual Quality	"A mí me encanto el diseño de la presentación en termino de los visuales de principio hasta fin. Me pareció amigable, como llamativa. Desde la primera vez que la vi, esa fue mi impresión inmediata, que era llamativa"
	Audio Quality	"Bueno, creo que la calidad pudiera ser un poco mejor. El volumen variaba dependiendo en unos slides donde el volumen estaba más alto y otros más bajos y la calidad "half and half" (participant said the portion in quotations in English)."
	Narrator's Voice	"No me molesta, me parece que habla claro"
Content	Complexity or unfamiliarity with language	"Si es la primera vez, puede ser que fuera un poco confuso o así, porque el lenguaje no fuera algo normal para eso, "you know" (participant said the portion in quotations in English)"<br>"Obviamente, también pienso que la persona tiene que tener un poquito, un poquito de conocimiento de lo que es la genética porque van a ver términos que a lo mejor no le van a hacer muy familiares"
	Easy to understand/ simple	"La presentación está sumamente bien redactada en términos de llevar en un lenguaje entendible mucha información que no es tan sencilla"<br>"Me parece que está bastante completa la información y me gusta que el lenguaje es simple, aun cuando la información que está dando es bastante detallada información en cuanto a cuáles son las causas y como funciona"<br>"Sí, claro. La información es clara y sencilla de entender"
	Value of information	"Yo creo que una de las cosas que recuerdo que se quedó conmigo era que aunque yo soy hombre, tengo y tengo hijos, incluido a una niña– creo que era útil para mí hacer el examen porque puede impactar. Me puede dar información para mi hija, aunque yo soy hombre"<br>"Este, lo cual fue bueno para mí estar interesada en este variante que obviamente ahora mismo no han sido estudiadas, hacía que no es, pero, haber visto la presentación me ayudo a entender que era lo que significaba eso [VUS]… Todavía no he tenido la cita con la doctora y eso es la semana que viene, pero por lo menos pude tener una idea de que era lo que yo estaba mirando cuando vi los resultados. Esto, yo creo… Yo creo que la presentación es sumamente valiosa"
Comprehension	Understanding of Hereditary Cancer	"Bueno, según entendí [cáncer] hereditario es menos posible. ¿Solo un 10%, verdad? Es menos posible cogerlo por herencia"<br>"Podría decirlo en palabras sencilla, hay información genética que podemos heredar y se pudiera o no desarrollar, pero que tenemos un riesgo es que tenemos una predisposición por dar esa información genética, sí. Como le dije, es una ruleta Rusa, podemos tener el gen y no nos dé [cáncer]. Poder tenerlo, y si nos da [cáncer]."
	Risk Perspective	"Pues, debido a mi historial familiar, yo tengo que tener un poquito más de cuidado de lo que tienen otras personas, aun cuando mis resultados de los genes específicos que se estudiaron, que se están estudiando, salieron negativos. Este, mi historial familiar pues en ese caso es el que predomina a sí que mi riesgo es un poquito más alto de lo normal"
Ge ne	Mode of Delivery	"Yo pienso siempre que flyers y pamphlets, ese tipo de cosas es algo que de verdad ayuda con asuntos médicos"

	"Mira, si hubiese como una entrevista, una persona [hablando] con otra, o probablemente un testimonio, como que, mira, yo salí negativa y mi mamá salió positiva, era positiva y mira, y hicimos el examen, y resulto ser que yo salí. Huh, uh, uh, me entiendes? Entonces ese mismo que tú explicaste, pero que una persona lo diga, entiende, yo te aseguro que me voy a quedar [prestando atención] mas."
Additional Information Needed	"Si a caso, indagar un poquito más en las cuestiones de los seguros, porque yo creo que eso es un área… no sé. A lo mejor si yo fuera la única que tuviese, pero yo creo que indagar un poquito más para darle más seguridad a las personas que si contemplan tener estos examines hechos en el futuro para darle más seguridad que ellos están protegidos"<br>"si la segunda parte dice las mutaciones pueden ser causadas por errores durante la división celular o pueden ser causadas por la exposición agentes en el medio ambiente que dañan el ADN … Aunque no sé si vas a explicar que son esos agentes después, pero quisas lo van a explicar después"
Access Barriers	"Por ejemplo,… Personas más mayores que van a necesitar un poquito de seguimiento (en términos de comprender conceptos)"<br>"Si lo ve alguien como nosotros, no le encontramos barreras pero para alguien que no pueda escucharlo, porque se le va a hacer difícil por lo menos escuchar lo que él [narrador dice] mientras leyendo creo que no, pero eso es tomando en consideración que esa comunidad vaya a acceder a este tipo o esta presentación"
Endorsement For General Public Use	"Me pareció muy educativa, y me pareció como algo que se debe compartir con más gente, no solo las personas que participan en este estudio, porque hay muchas veces que a veces hasta en programas de televisión y eso hacen chistes al respecto, pero que muchas veces las personas no entienden lo que significa hasta los conceptos más básicos, hasta negativo o positivo"<br>"Es buena idea que otra persona antes de pasar por este proceso viera esta presentación. Es bien informativa"

Table S2: Themes from semi-structured interviews related to possible testing outcomes with quotes in Spanish

Themes	Exemplar Quotes	Interpretive conclusions
Testing benefits family members	*"Esos que salen positivos con el resultado en el BRCA que estuvimos hablando indica que cuando un hombre, aunque no lo tenga, que lo lleva de su madre o alguien, puede afectar a la niña mía que puede tener mayores posibilidades para obtenerlo [cáncer]" ;* <br>*"… los resultados ayudan a la familia"*	Suggests understanding that even if they never developed cancer, their children could be at risk if they were to inherit a mutation in a cancer predisposing gene.
Importance of cancer family history	*"Este tipo de cáncer esporádico, hereditario, etc, de las pruebas genéticas de mutaciones, que cuando sale positivo, que cuando sale un VUS, cuando es negativo, pero siempre tomando en consideración los antecedentes familiares de cáncer hasta el final"*	Suggests understanding of the importance of family history in considering cancer screening, even without a positive genetic test result.
Responses to positive genetic test results	*"Destruido. Me sentiría mal. Me sentiría como preocupado o muy preocupado"*	Demonstrates an emotional response of concern or worry about the results which is appropriate.
	"Luchar para poder tener vida y ver cuan posible es la recuperación adecuada"	Suggests a potential lack of clarity in the meaning of a positive test result, and viewing a positive genetic test result similar to being given a cancer diagnosis.
	"Pues, yo creo que bastante preparada, verdad? Este en el sentido de saber cuál es el próximo paso y además de discutirlo con el médico primario, compartirlo con familiares o progenie. Mis hijos y en el momento que los tenga dejarle saber que esto está comprobado genéticamente o tienes que estar "aware" (participant said word in quotations in English)"	Demonstrates understanding of next steps based on positive results, including seeking prevention, speaking with doctors, and sharing the information with family members.
Responses to negative genetic test results	*"Confundida contigo porque si la recibo negativo y yo soy positiva para cáncer de seno. Este, no sé, por una parte, puede ser. Si yo me saco eso, la prueba genética negativa y yo tengo cáncer, o si sale positiva para cáncer como mis hijas tuvieran eso. O sea, cómo? "*	Suggests a lack of understanding regarding the meaning of a genetic test result.
	"Fantastic, great, awesome (participant said this portion in English). Estuviera contento. Me sentiría más confiado, pero eso no me quita las ganas de chequearme a cada rato"	Demonstrates understanding that negative results do not completely eliminate all cancer risks, and that prevention methods and check-ups would still be sought out
Responses to VUS genetic test results	*"Si es como lo que entendí del video, es que ocurrió una mutación al azar, que no es hereditario. No es algo que heredamos de mamá o papá, pero que fue una mutación que ocurrió al azar en el cuerpo y pues ese cambio genético se encontró en la prueba, pero no se ve, no se determina que viene de mamá o que viene de papá"*	Suggests trying to differentiate result from a positive result, yet misunderstanding that a VUS may or may not be inherited.
	"Pues como quiera hay incertidumbre porque este como hay que dejarse llevar por los antecedentes familiares de cáncer también. No, es que estás excluido o que estás exonerado a un 100%"	Demonstrates understanding that increased risks of cancer may exist based on family history thus seeking clinical advice and screenings are still important

	"Se que me sentiría igual como que si me hubiese dado negativo"	Demonstrates reaction to VUS and negative results would be similar which is appropriate given that most VUS results are ultimately reclassified as negative.
